# Biomarkers of delirium in critically ill patients

**DOI:** 10.1186/cc9760

**Published:** 2011-03-11

**Authors:** M Van den Boogaard, L Schoonhoven, K Quinn, M Kox

**Affiliations:** 1 Radboud University Nijmegen Medical Centre, Nijmegen, the Netherlands; 2 Scientific Institute for Quality of Healthcare, Radboud University Nijmegen Medical Centre, Nijmegen, the Netherlands; 3 Departments of Anesthesia and Critical Care, St Michael's Hospital, Toronto, Canada

## Introduction

Delirium occurs frequently in critically ill patients and is associated with disease severity and infection. Although several pathways for delirium have been described, biomarkers associated with delirium in ICU patients are unknown. We examined differences in levels of several biomarkers in matched delirious and nondelirious patients admitted to the ICU.

## Methods

Delirium in adult ICU patients was diagnosed using the Confusion Assessment Method-ICU (CAM-ICU). Delirious and nondelirious patients were meticulously matched for age, APACHE II score, presence or absence of infection or SIRS criteria, and length of ICU stay at the moment of blood withdrawal. Neurology and trauma patients were excluded. Within 24 hours after the development of delirium, blood was drawn for determination of biomarkers. Covariate analyses were performed using the C-reactive protein (CRP) level to adjust for severity of infection.

## Results

Fifty delirious and 50 nondelirious ICU patients were included. Levels of TNFα, IL-6, IL-8, MIF, IL-1ra, IL10, MCP-1, PCT, cortisol, and the brain-specific protein amyloid-β truncated-40 were significantly higher in delirious ICU patients. The ratio of amyloid-β 42/40 and truncated 42/40 were significantly lower in delirious compared with nondelirious ICU patients, suggesting more deposition of amyloid-β in the brain. In a multivariate logistic analysis adjusted for severity of infection, levels of TNFα, IL-8, IL-1ra, IL-10, MCP-1 and PCT were significantly higher in the delirious group. The ratio of amyloid-β 42/40 and truncated 42/40 (both *P *= 0.056), IL-6 (*P *= 0.057) and MIF (*P *= 0.081) tended to be different in delirious ICU patients.

## Conclusions

In ICU patients, delirium is associated with significantly increased concentrations of TNFα, IL-8, IL-1ra, IL-10, MCP-1, PCT and a decreased ratio of amyloid-β 42/40, even after adjusting for severity of infection. We conclude that several proinflammatory and anti-inflammatory cytokines, PCT and amyloid-β are associated with delirium in ICU patients, and could therefore serve as possible biomarkers.

